# TRAP1: A Metabolic Hub Linking Aging Pathophysiology to Mitochondrial *S*-Nitrosylation

**DOI:** 10.3389/fphys.2020.00340

**Published:** 2020-04-29

**Authors:** Fiorella Faienza, Salvatore Rizza, Paola Giglio, Giuseppe Filomeni

**Affiliations:** ^1^Department of Biology, Tor Vergata University of Rome, Rome, Italy; ^2^Redox Signaling and Oxidative Stress Group, Danish Cancer Society Research Center, Copenhagen, Denmark

**Keywords:** tumor necrosis factor receptor-associated protein 1 (TRAP1), *S*-nitrosylation, mitochondria, aging, cancer, Parkinson's disease

## TRAP1: A Chaperone With Manifold Metabolic Effects

Tumor necrosis factor receptor-associated protein 1 (TRAP1), also known as heat shock protein 75 (Hsp75), is the mitochondrial member of the Hsp90 family of molecular chaperones, which acts as a key regulator of mitochondrial homeostasis and bioenergetics (Hoter et al., 2018). At variance with the other members, TRAP1 (i) shows a marked asymmetric conformation; (ii) can use both Ca^2+^ and Mg^2+^ as cofactors; and (iii) aids substrate folding without co-chaperone assistance, this making it unique among Hsp90 family members (Lavery et al., 2014; Masgras et al., 2017b; Elnatan and Agard, 2018).

To date, few TRAP1 clients have been discovered, most of which are proteins involved in different mitochondrial functions, such as apoptosis and metabolism control (Hoter et al., 2018). TRAP1 exerts a protective role in mitochondria and is able to prevent oxidative stress-induced cell death through the inhibition of the permeability transition pore (PTP) opening (Matassa et al., 2018). This effect can be both direct, through the inhibition of cyclophilin D (CypD), and indirect, through the modulation of reactive oxygen species (ROS) concentration (Amoroso et al., 2014; Matassa et al., 2018). Such ability is intimately linked to TRAP1 role in metabolism, i.e., the control of electron flow along the respiratory complexes of the electron transport chain (ETC) (Masgras et al., 2017b). In particular, TRAP1 is able to downregulate both Complex II and Complex IV activities, which occurs in concert with phosphorylation events mediated by mitochondria-localized kinases (Yoshida et al., 2013; Masgras et al., 2017a). This feature represents a key intersection point in TRAP1-mediated control of metabolism since Complex II takes part in the ETC but also acts as the succinate dehydrogenase (SDH) within the tricarboxylic acid (TCA) cycle (Sciacovelli et al., 2013). SDH catalyzes the conversion of succinate into fumarate; therefore, its inhibition causes an imbalance in the relative concentration of these metabolites, i.e., an increase of succinate (Sciacovelli et al., 2013; Rizza et al., 2016). It has been demonstrated that this accumulation enables the release of the hypoxia-inducible factor 1α (HIF1α) inhibition. This event stabilizes the active dimer HIF1α/β, leading to the activation of HIF1-mediated transcription of genes coding for glycolytic enzymes, and for BCL2 and adenovirus E1B 19-kDa-interacting protein 3 (Bnip3), which mediates mitochondrial selective removal by autophagy (mitophagy) (Singh et al., 2017; Zhang et al., 2018). In such a way, TRAP1 is able to rewire metabolism by downregulating mitochondrial oxidative phosphorylation (OXPHOS) and promoting glycolysis, a feature resembling the so-called “Warburg effect” distinctive of cancer cells (Yoshida et al., 2013). As a result, OXPHOS downregulation is associated with limited ROS production, increased mitochondrial tolerance to oxidative stress, and protection from apoptosis (Masgras et al., 2017b; Matassa et al., 2018).

## TRAP1 Is a Target of *S*-Nitrosylation

*S*-nitrosylation is a redox posttranslational modification of cysteine residues induced by nitric oxide (NO) (Rizza and Filomeni, 2016). It is dynamically regulated by the amount of NO produced by NO synthases (NOSs) and exchanged between nitrosothiols (SNOs) and free sulfhydryls (SHs), as well as by the ability of denitrosylases to reduce SNO groups. *S*-nitrosoglutathione reductase (GSNOR) is the prototype of this class of enzymes. It contributes to regulate the levels of *S*-nitrosylated proteins (PSNOs) (Rizza and Filomeni, 2017); therefore, conditions of GSNOR deficiency are associated with a general increase of PSNOs. Recently, it has been demonstrated that *Gsnor*-null cells are characterized by mitochondrial dysfunction and metabolic changes (Rizza et al., 2018). Moreover, it has been reported that GSNOR-downregulating hepatocellular carcinoma (HCC) cells show reduced levels of TRAP1 (Rizza et al., 2016). Mass spectrometry analyses indicated that this phenomenon is associated with selective nitrosylation of Cys501 in TRAP1, and cell biology experiments provided the evidence that this modification induces loss of stability of TRAP1 and its accelerated degradation *via* the proteasome (Rizza et al., 2016).

Besides this regulation, it has also been very recently reported that *S*-nitrosylation of Cys501 produces a decrease of TRAP1 ATPase activity likely through an allosteric mechanism (Faienza et al., 2020). ATPase assays, together with molecular dynamics simulations, indicate that *S*-nitrosylation negatively impacts TRAP1 activity *i*) *directly*, through intra- and inter-protomer long-range communication events that exert distal effects on the active site, and *ii*) *indirectly*, through the regulation of open-to-close state transition, which is crucial for this class of chaperones to complete the ATPase cycle (Faienza et al., 2020). Cells expressing a mutant form of TRAP1, in which Cys501 is substituted by a serine (C501S), are more resistant to mitochondrial toxins (Rizza et al., 2016) and to staurosporine-induced apoptosis (Faienza et al., 2020), suggesting that *S*-nitrosylation of Cys501 impacts TRAP1 biology. Integrating these pieces of evidence, it is reasonable to propose that *S*-nitrosylation-induced degradation of TRAP1 is a direct consequence of its loss of activity.

## Effects of TRAP1 *S*-Nitrosylation in Aging

Various lines of evidence indicate that GSNOR downregulation, or loss, correlates with cell senescence and mammalian aging (Rizza et al., 2016, 2018). Based on what above reported, this allows speculating that TRAP1 expression could be also involved, or play a role, in aging physiopathology. Given the function of TRAP1 as mitochondrial chaperone and its importance in mitochondrial proteostasis, this hypothesis is credible. Aging is, indeed, accompanied by a progressive decline of mitochondrial functions and turnover, which, according to the mitochondrial free radical theory of aging, is considered causative of a number of age-associated pathologies, such as neurodegenerative diseases (Akbari et al., 2019) and cancer (López-Otín et al., 2013).

Studies conducted in fruit flies have revealed that TRAP1 overexpression benefits insect health by increasing fertility and locomotor ability (Baqri et al., 2014). The positive effects induced by TRAP1 overexpression in *Drosophila* are directly related to its ability to modulate heat and oxidative stress resistance through the regulation of mitochondrial proteostasis and the activation of the mitochondrial unfolded protein response (UPR^mt^) (Baqri et al., 2014; Gumeni and Trougakos, 2016). The loss of proteostasis is a signature of aging and is closely related to chaperone functions (López-Otín et al., 2013; Sala et al., 2017). Interestingly, this is exacerbated during aging by PSNOs accumulation.

### Parkinson's Disease

Due to the role of TRAP1 in the maintenance of mitochondrial homeostasis, it is conceivable that any dysregulation of its expression/activity may be associated with the onset of diseases related to mitochondrial dysfunctions. In agreement with this assumption, negative modulation of TRAP1 protein levels has been frequently reported in *in vitro* and *in vivo* models of Parkinson's disease (PD) (Pridgeon et al., 2007; Costa et al., 2013; Zhang et al., 2013; Fitzgerald et al., 2017). In particular, it has been demonstrated that ectopic expression of TRAP1 is able to counteract dysfunctional phenotypes in PD models, such as those induced by α-synuclein overexpression, preventing, or attenuating, mitochondrial defects and apoptosis (Butler et al., 2012; Fitzgerald et al., 2017; Hoter et al., 2018). Moreover, in *Drosophila* and mammalian models of PD, it has been reported that TRAP1 overexpression fully rescues mitochondrial impairments associated with phosphatase and tensin homolog (PTEN)-induced kinase 1 (PINK1) loss of function, but only partially those induced by Parkin mutation (Costa et al., 2013; Zhang et al., 2013), suggesting that TRAP1 acts downstream of PINK1 and in parallel to Parkin. Interestingly, TRAP1 loss of function in *Drosophila* phenocopies the effects induced by PINK1 deficiency, including mitochondrial and locomotor activity defects (Costa et al., 2013).

TRAP1 ability to rescue PINK1 deficiency goes through TRAP1 phosphorylation by PINK1 (Pridgeon et al., 2007), which enhances its ability to inhibit oxidative stress and to protect neuronal cells from death (Fitzgerald et al., 2017; Hoter et al., 2018). Along the same line, a homozygous loss-of-function mutation of TRAP1, caused by a premature stop codon, has been identified in a sporadic PD patient. Studies performed in fibroblasts obtained from this patient revealed different molecular phenotypes distinctive of mitochondrial dysfunction, e.g.: i) high ROS levels; ii) impaired UPR^mt^; iii) loss of mitochondrial transmembrane potential; iv) enhanced susceptibility to mitophagy and apoptosis (Fitzgerald et al., 2017). However, debates on the methods used in this study to perform genome datasets analyses, together with the low frequency of this mutation, call into question the general relevance of this finding in PD research (Fitzgerald et al., 2018; Gaare et al., 2018).

Mitochondrial functions, as well as PINK1 and Parkin activities, are affected by conditions of excessive *S*-nitrosylation, which are a distinctive signature of PD brains. How much this is a cause or consequence of PD, or contributes in a positive feedback loop to the establishment of the pathological state, is still a matter of debate. What is known is that *S*-nitrosylation at Cys658 in PINK1 inhibits PINK1 kinase activity and, in turn, compromises mitophagy and viability of PD cellular models (Oh et al., 2017). Likewise, Parkin *S*-nitrosylation has been proposed to affect Parkin E3-ligase activity, thereby causing accumulation of misfolded proteins and damaged mitochondria (Chung et al., 2004; Yao et al., 2004; Nakamura and Lipton, 2013; Ozawa et al., 2013). Based on these lines of evidence, it is conceivable that *S*-nitrosylation negatively affects TRAP1—and, in turn, mitochondrial homeostasis, metabolism, and apoptosis—at different levels, acting both directly (at Cys501) and indirectly (through the inhibition of its upstream regulator PINK1, or Parkin). Whatever the mechanism is, the hypothesis that TRAP1 *S*-nitrosylation plays any role in neuronal injury distinctive of PD and—at least in principle—in other neurodegenerative diseases is realistic and claims for further investigations, e.g., to identify TRAP1 clients downstream of PINK1 and understand their regulation following PINK1 and TRAP1 *S*-nitrosylation.

### Cancer

By several reasons, cancer can be considered another age-related disease. Aging is, indeed, a major risk factor in cancer development. Former studies, aimed at investigating the role of TRAP1 in cancer, highlighted that it is overexpressed in several tumor tissues (if compared with peer non-tumor counterparts) and suggested that this phenomenon was linked to TRAP1 ability to rewire metabolism and favor the “Warburg effect” (Sciacovelli et al., 2013; Masgras et al., 2017a,b; Matassa et al., 2018). These assumptions have been redefined in the last few years as: *i*) some tumors are not fully glycolytic but, depending on environmental availability of nutrients, can interchangeably use OXPHOS to sustain their accelerated growth, and *ii*) TRAP1 has been found differentially expressed in different tumors—and in different stages within the same tumor—in order to adjust metabolism to tumor cell needs (Matassa et al., 2018).

A context-dependent effect in cancer is common also to *S*-nitrosylation, with NO being considered a “Janus faced” molecule in tumorigenesis, due to its ability to both promote or inhibit cancer cell growth (Di Giacomo et al., 2012). From this angle, TRAP1 nitrosylation at Cys501 can play the role of tuner of TRAP1 oncogenic role. Structurally, Cys501 is placed close to Ser511 and Ser568, which are the two residues indicated as preferential targets of phosphorylation by the extracellular signal-regulated kinase 1 and 2 (ERK1/2) (Masgras et al., 2017a). ERK1/2 binding and phosphorylation stimulate the formation of TRAP1/SDH multimeric complex and enhance the inhibitory activity of TRAP1 on SDH (Masgras et al., 2017a), thereby positively contributing to the TRAP1-induced metabolic reprogramming of cancer cells. Based on this evidence, it is reasonable to hypothesize that *S*-nitrosylation might interfere with ERK1/2-mediated phosphorylation, or, *vice versa*, phosphorylation impedes Cys501 accessibility to the solvent, and its propensity to be modified by NO. Whatever is the direction of this interplay, it is plausible that Cys501 nitrosylation and Ser511/568 phosphorylation are two mutually exclusive posttranslational modifications of TRAP1. How much this cross talk impacts TRAP1 allosteric regulation and its capability to act as a metabolic hub rather than its antioxidant or anti-apoptotic roles still wait to find answer. Nevertheless, given the effects of *S*-nitrosylation on TRAP1 oncogenic properties, the use of NO donors can be hypothesized as useful approaches in combined treatments aimed at selectively killing chemoresistant tumors where TRAP1 is overexpressed and contributes to apoptosis resistance (Costantino et al., 2009).

### Is TRAP1 an Epigenetic Modulator of Aging and Aging-Related Diseases *via S*-Nitrosylation?

**Figure 1:**
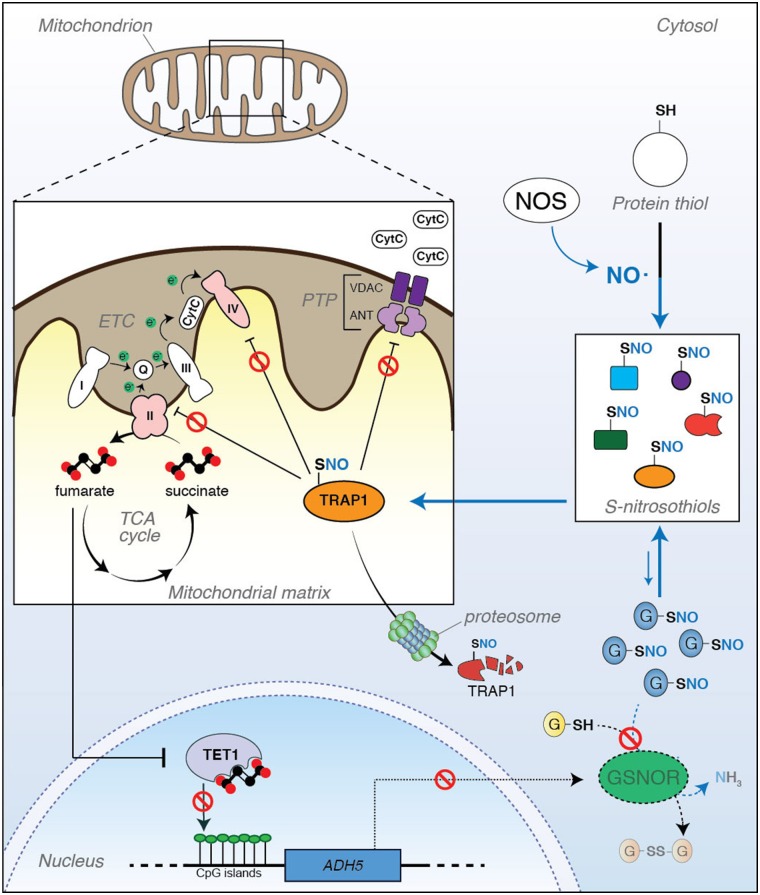
Tumor necrosis factor receptor-associated protein 1 (TRAP1) is a metabolic hub linking *S*-nitrosylation to aging. (*Right*) *S*-nitrosylation is a posttranslational modification induced by nitric oxide (NO). It regulates the activity, localization, stability, and functions of a series of cysteine (SH)-containing proteins that, upon reaction with NO, become nitrosylated (*S*-nitrosothiols, or SNOs). Protein *S*-nitrosylation extent does not only depend on the rate of NO synthesis by NO synthase (NOS), but it is also controlled by the ability of a class of enzymes (denitrosylases) to catalyze the SNO-to-SH reduction. *S*-nitrosoglutathione reductase (GSNOR) is a well-known and, probably, the best characterized example of denitrosylase so far identified. However, it does not directly react with protein-SNOs. Actually, GSNOR catalyzes the reduction of *S*-nitrosoglutathione (GSNO) to glutathione (GSH). GSNO levels are in equilibrium with protein-SNOs through a spontaneous exchange reaction called *trans*-nitrosylation; therefore, by controlling GSNO levels, GSNOR indirectly regulates the extent of protein *S*-nitrosylation. (*Left*) It has been recently reported that TRAP1 undergoes S-nitrosylation, this impacting its stability. Inside the mitochondrion, TRAP1 antagonizes PTP opening and downregulates OXPHOS through the inhibition of Complexes II and IV of the electron transport chain (ETC), both these events preventing cytochrome *c* (CytC) release and apoptosis. However, when *S*-nitrosylated at Cys501, TRAP1 activity decreases, this probably inducing its degradation by the proteasome with a consequent increase in apoptosis susceptibility and Complex II activity. It can be speculated that this condition might result in a disbalance of succinate-to-fumarate ratio (i.e., an accumulation of fumarate levels), which can impact ten-eleven translocation (TET)1-dependent epigenetic activity and, in turn, produce a hyper-methylation of CpG islands in the promoter of several genes. Recently, it has been reported that *ADH5* (the gene coding for GSNOR) is among those genes which are epigenetically controlled by TET1, and its silencing contributes to cell senescence and mammalian aging. In this scenario, a new role of TRAP1 as an epigenetic factor of aging can be hypothesized, with *S*-nitrosylation acting as a modulatory event of this loop of regulation.

As previously described, TRAP1-mediated inhibition of SDH generates an accumulation of succinate that modulates HIF1 transcriptional activity. This phenomenon depends on the inhibitory effects that succinate exerts on α-ketoglutarate-dependent dioxygenases, a group of enzymes that hydroxylate different substrates, i.e., HIF1α (Laukka et al., 2016). This family of enzymes also includes several epigenetic regulators, such as histone demethylases and 5-methylcytosine (5meC) hydroxylases [also referred to as ten-eleven translocation (TET) proteins] (Xiao et al., 2012). Epigenetic modifications, such as those induced by alterations of TETs, have been included in the hallmarks of aging (López-Otín et al., 2013). Concerning this aspect, we have recently discovered a functional association between TET1 and GSNOR expression (Rizza and Filomeni, 2018; Rizza et al., 2018), with both proteins decreasing during aging. However, many aspects, mostly those aimed at understanding i) if the relationship between TET1 and GSNOR is biunivocal and regulated by a feedback loop; ii) how TET1/GSNOR signaling axis is initiated; iii) how it is fueled and kept sustained during life span, remain still unanswered. In regard to this, TRAP1 could represent the missing link underlying the close relationship between TET1 and GSNOR in aging. Namely, by affecting SDH activity, TRAP1 decrease by *S*-nitrosylation could result in a disbalance of succinate-to-fumarate ratio, which, consequently, i) inhibits TET1 activity, ii) leads to an increase in CpG island methylation in several promoters, and iii) causes a decrease of protein expression (as, indeed, observed for GSNOR). Therefore, consistent with the establishment of a positive feedback loop, *S*-nitrosylation could target TRAP1 and keep TET1 inactive to initiate and sustain epigenetic silencing of GSNOR expression (Figure 1).

## Concluding Remarks

Oxidative damage to cells and tissues has always been considered a major cause of aging. Being the main ROS production site inside the cells, mitochondria are usually indicated as the principal source of oxidative stress, with their dysfunction being causative of (or at least contributing to) aging and age-related diseases (Akbari et al., 2019).

Recently, it has been proposed that *S*-nitrosylation plays a role in the onset of aging. Several mitochondrial proteins found to be associated to neurodegeneration (e.g., Drp1, PINK1, Parkin) are, indeed, target of *S*-nitrosylation, and recent findings about the deleterious effects of GSNOR deficiency on mitochondrial functions and mammalian longevity further support these observations (Rizza et al., 2018). In this scenario, TRAP1 modulation by excessive *S*-nitrosylation could represent a new regulatory mechanism involved in aging and age-related pathophysiology, other than a means to reprogram cell metabolism.

In this Opinion, we have attempted to provide food for thought and elaborate on the potential impact of TRAP1 *S*-nitrosylation in mitochondrial physiology, with relevance to aging and age-related diseases (e.g., cancer and neurodegeneration), which are, *de facto*, pathological states associated with mitochondrial dysfunctions.

## Author Contributions

FF and GF wrote the paper. SR and FF conceived and drew up the figure. PG and all authors discussed, corrected, and critically read the paper.

## Conflict of Interest

The authors declare that the research was conducted in the absence of any commercial or financial relationships that could be construed as a potential conflict of interest.

## Abbreviations

*5meC*: 5-methylcytosine
*Bnip3*: BCL2 and adenovirus E1B 19-kDa-interacting protein 3
*CypD*: cyclophilin D
ERK1/2: extracellular signal-regulated kinase 1 and 2
*GSNOR*: S-nitrosoglutathione reductase
*HCC*: hepatocellular carcinoma
*HIF1*α: hypoxia inducible factor 1α
*Hsp*: heat shock protein
*NO*: nitric oxide
*NOS*: nitric oxide synthase
*OXPHOS*: oxidative phosphorylation
*PINK1*: PTEN-induced kinase 1
*PSNOs*: S-nitrosylated proteins
*PTP*: permeability transition pore
*ROS*: reactive oxygen species
*SDH*: succinate dehydrogenase
*TCA*: tricarboxylic acid
*TET*: ten-eleven translocation
*TRAP1*: tumor necrosis factor receptor-associated protein 1
*UPR*^*mt*^: mitochondrial unfolded protein response.
